# Prevalence and clinical associations of mitral and aortic regurgitation in patients with aortic stenosis

**DOI:** 10.1111/echo.15503

**Published:** 2022-12-15

**Authors:** Petro Gjini, Jodie F. Kenes, Mahesh Chandrasekhar, Ross Hansen, Ajay Dharod, Stephen C. Smith, Min Pu, Bharathi Upadhya, Richard Brandon Stacey

**Affiliations:** ^1^ Evans Department of Medicine Boston University School of Medicine Boston Massachusetts USA; ^2^ Dearborn Cardiology Dearborn Michigan USA; ^3^ Moses Cone Hospital Cone Health Greensboro North Carolina USA; ^4^ Section of Cardiovascular Medicine Department of Internal Medicine Wake Forest University School of Medicine Winston‐Salem North Carolina USA; ^5^ Division of Cardiology Albert Einstein School of Medicine New York New York USA

**Keywords:** aortic regurgitation, aortic stenosis, echocardiography, mitral regurgitation, valvular heart disease, volume overload

## Abstract

**Background:**

Most guidelines directing clinicians to manage valve disease are directed at single valve lesions. Limited data exists to direct our understanding of how concomitant valve disease impacts the left ventricle (LV).

**Methods:**

We identified 2817 patients with aortic stenosis (AS) from the echocardiography laboratory database between September 2012 and June 2018 who had a LV ejection fraction (EF) ≥50%. LV mass, LV mass index, LV systolic pressure (systolic blood pressure + peak aortic gradient). Covariates were collected from the electronic medical record. Multi‐variate analysis of covariance was used to generate adjusted comparisons.

**Results:**

Our population was 66% female, 17% African‐American with a mean age of 65 years. Of note, 7.3% were noted to have significant (moderate/severe) aortic regurgitation (AR), and 11% had significant (moderate/severe) mitral regurgitation (MR). Adjusting for covariates at different levels, significant MR had a much stronger association with heart failure compared to those with significant AR (*p* < .001 vs. *p* = .313, respectively) at all levels of adjustment. Both significant mitral and AR exhibited an association with increasing left ventricular mass, even with adjustment for baseline demographics and clinical features (*p* < .001 vs. *p* = .007, respectively).

**Conclusion:**

In patients with AS, 16% also experience at least moderate MR or AR. Further, significant MR has a stronger association with heart failure than significant AR, even though both increase left ventricular mass. Those with moderate AS and significant MR or AR experience similar or higher levels of heart failure compared to severe AS without regurgitation. Mixed valve disease merits further studies to direct longitudinal management.

## 1 INTRODUCTION

As world populations continue to age, clinically significant aortic stenosis (AS) will continue to become more prevalent.^1^, ^2^, ^3^ Consequently, identifying AS becomes even more important. The guidelines provide an evidence‐based approach for diagnosis and management, but some gaps remain to be addressed when mixed valvular diseases are present.^4^, ^5^, ^6^ In addition, the presence of either aortic or mitral regurgitation (MR) may make it more difficult to accurately assess the severity of AS.^7^, ^8^

In situations without AS, MR leads to volume overload and left ventricular dilatation,^9^, ^10^, ^11^ and aortic regurgitation (AR) leads to both pressure and volume overload, which manifest as left ventricular dilatation with thickened walls.^12^, ^13^, ^14^ Interestingly, MR is less well tolerated than AR and leads to earlier development of heart failure.^15^

However, the effect of these concomitant lesions with AS on LV remodeling is not well elucidated. In addition, the natural history of mixed valvular disease is not well known.^16^, ^17^ This manuscript seeks to add to our knowledge of the prevalence and potential clinical risk of concomitant valve disease and provide justification for a more prospective investigation to direct clinical management. These analyses also seek to compare the initial remodeling seen in those with AS with and without either mitral or AR.

## 2 METHODS

The Wake Forest University School of Medicine Institutional Review Board (IRB) reviewed and approved this research study. With this being a retrospective study of data obtained during routine clinical care, the IRB granted a waiver of informed consent. After obtaining Institutional Review Board approval, patients shown to have mild, moderate, or severe AS with a preserved left ventricular ejection fraction (EF) >50% were identified in the Wake Forest School of Medicine echocardiography database. Those with a left ventricular EF <50% were excluded because of the difficulty in accurately assessing the degree of AS in that population. Between September 2012 and June 2018, a total of 2817 patients were identified with reports that included the above parameters, and these clinical cases comprised the study population. Clinical and demographic data were extracted from the electronic medical records.

### 2.1 Echocardiography

The echocardiographic measurements included left ventricular dimensions, left ventricular ejection fraction (LVEF; determined with a modified biplane Simpson's method), the transvalvular gradients as determined by the simplified Bernoulli equation, and aortic valve area using the continuity equation.^18^ LV mass index was calculated using the Devereux formula.^19^, ^20^

### 2.2 Aortic valve disease

Doppler echocardiography data were used to categorize patients by aortic valve disease severity. Severe AS was defined as an AVA ≤1.0 cm^2^, moderate AS was defined as an AVA 1.0–1.5 cm^2^, and mild AS was defined as an AVA >1.5 cm^2^.^5^, ^6^ For classification purposes, AVA was used preferentially because of the potential impact of concomitant aortic or MR on transaortic gradients.^7^, ^8^

### 2.3 Valvular regurgitation

Valvular regurgitation was assessed according to the American Society of Echocardiography (ASE) valvular regurgitation guidelines and classified by one of our clinical echocardiographic faculty as reflected in their finalized echocardiogram report.^4^ Each interpreter was either a level‐II or level‐III trained echocardiographer with >80% having passed the National Board of Echocardiography. For analytical purposes, the valve regurgitation was divided into none, mild, moderate, or severe. When speaking of moderate and severe regurgitation together, the term “significant” is used.

### 2.4 Heart failure

Heart failure was captured by identifying those who were diagnosed with heart failure by a healthcare provider in their past medical history in the electronic medical record.

### 2.5 Statistical analyses

Results were expressed as mean ± SD or percentages. Differences between patient groups were analyzed using ANOVA for continuous variables, and the chi‐square test or Fisher's exact test for categorical variables, as appropriate. All statistical analyses were performed using SAS JMP 16.1 (Copyright by SAS Institute, Inc.)

To adjust for covariates, a graded model‐based approach was employed. For the relationship between left ventricular mass and AS as defined by AVA, an ANCOVA approach was used. Model I adjusted for AR, MR, body surface area, and peak aortic valve systolic pressure gradient, and Model II performed further adjustment for age, race, and sex. For model III, additional adjustments were made for clinical variables, such as coronary artery disease, hypertension, diabetes mellitus, and chronic kidney disease.

For the relationship between heart failure and AS as defined by AVA, a logistic regression approach was used. Model I adjusted for AR, MR, body surface area, and peak aortic valve systolic pressure gradient, and Model II performed further adjustment for age, race, and sex. For model III, additional adjustments were made for clinical variables, such as coronary artery disease, hypertension, diabetes mellitus, and chronic kidney disease.

## 3 RESULTS

After excluding those with a left ventricular EF less than 50%, prosthetic valves, endocarditis, or missing data, our study population consisted of 2817 patients with AS. Their characteristics by AS severity are shown in Table 1. Both the moderate and severe AS groups were older, had a higher prevalence of men, and had a lower prevalence of African Americans. In addition, both groups also had a higher prevalence of coronary artery disease and heart failure. Finally, both moderate and severe AS groups experienced higher rates of mitral and aortic valve regurgitation.

**TABLE 1:** An asterisk (*) indicates that it is statistically different from mild aortic stenosis (*p <* .05)

	Mild aortic stenosis (*n* = 1500)	Moderate aortic stenosis (*n* = 723)	Severe aortic stenosis (*n* = 594)
Age (years)	60.8 ± 17.9	67.9 ± 16.1*	69.1 ± 15.8*
Female (%)	72.3	62.5	56.7
African‐American (%)	19.4	16.1	12.6
Diastolic BP (mmHg)	70.8 ± 14.0	69.8 ± 14.8	67.5 ± 16.4*
Systolic BP (mmHg)	137.9 ± 28.5	141.5 ± 30.9*	135.6 ± 32.5
Height (cm)	165.6 ± 50.6	168.9 ± 94.3	162.7 ± 30.2
Weight (kg)	79.7 ± 22.6	79.4 ± 25.0	78.9 ± 25.9
Aortic root diameter (cm)	2.9 ± 0.4	3.1 ± 0.5*	3.1 ± 0.6*
Left atrial volume index (ml/m^2^)	29.2 ± 5.1	34.2 ± 5.5	35.0 ± 6.2
LV EF (%)	61.9 ± 6.8	62.4 ± 6.9	63.0 ± 7.4
LV EDV index (ml/m^2^)	51.7 ± 17.0	52.9 ± 18.8	50.5 ± 20.5
Max press gradient (mmHg)	18.1 ± 7.6	36.2 ± 10.1*	58.1 ± 9.8*
Mean press gradient (mmHg)	8.2 ± 5.2	27.3 ± 6.1*	39.2 ± 7.3*
Stroke volume index (ml/m^2^)	37.3 ± 11.3	38.1 ± 10.8	34.2 ± 15.4*
Moderate or severe MR (%)	8.2	13.2*	15.0*
Moderate or severe AR (%)	4.6	10.0*	11.1*
Coronary Artery Disease (%)	20.9	29.4*	29.5*
Diabetes mellitus (%)	28.6	32.5	32.0
Chronic Kidney Disease (%)	20.9	29.4*	29.5*
Heart failure (%)	11.4	16.2*	19.2*
Hypertension (%)	64.6	74.3*	74.8*
Current tobacco use (%)	8.1	7.2	6.6

Abbreviations: AR = Mitral Regurgitation; EF, ejection fraction; LV, left ventricle; MR, mitral regurgitation.

**FIGURE 1:**
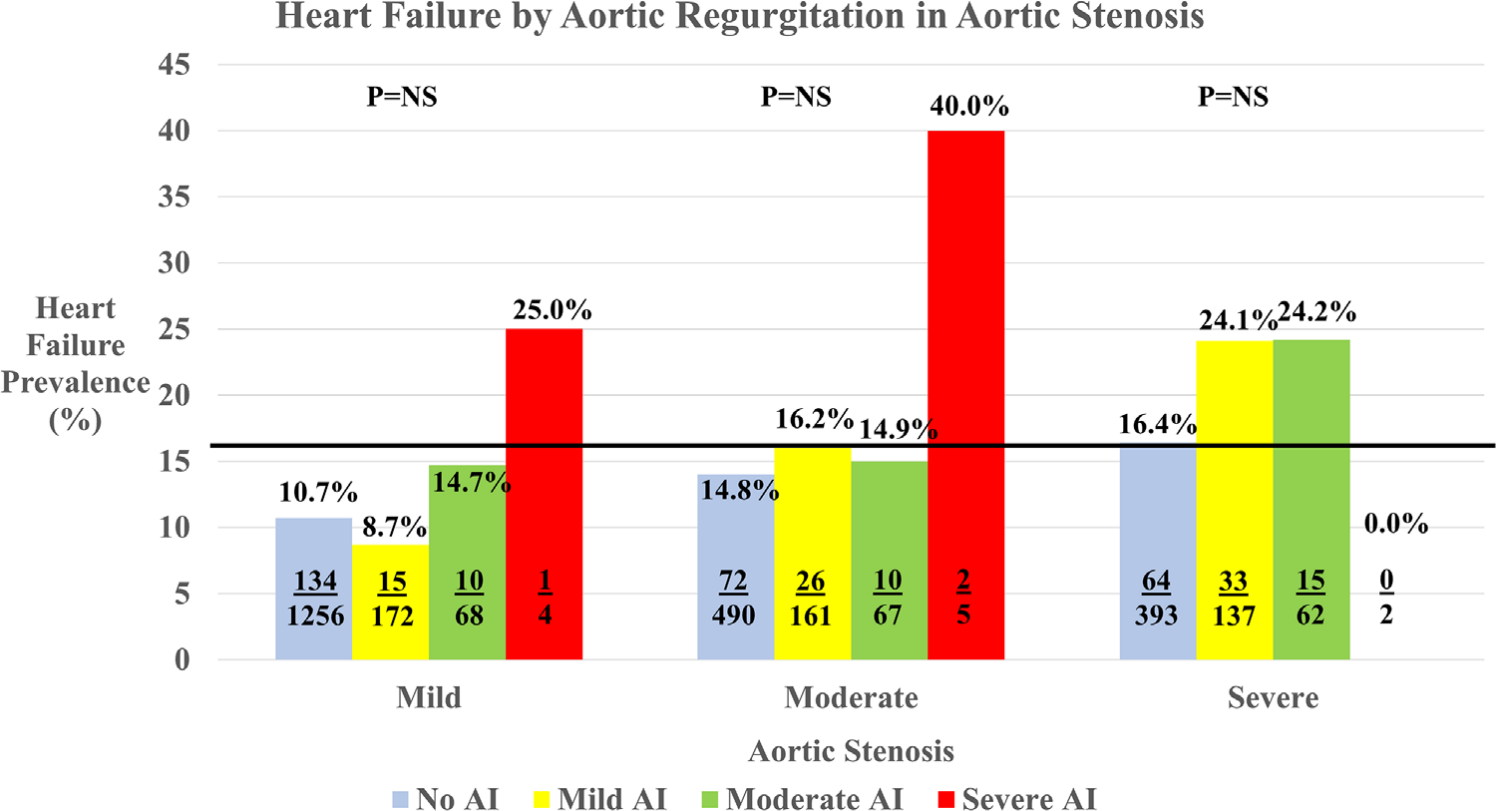
Heart failure prevalence by aortic regurgitation in aortic stenosis (AS). The p value represents testing for significant difference within the differing degrees of AS. The solid line indicates the prevalence of heart failure in severe AS without regurgitation

**FIGURE 2:**
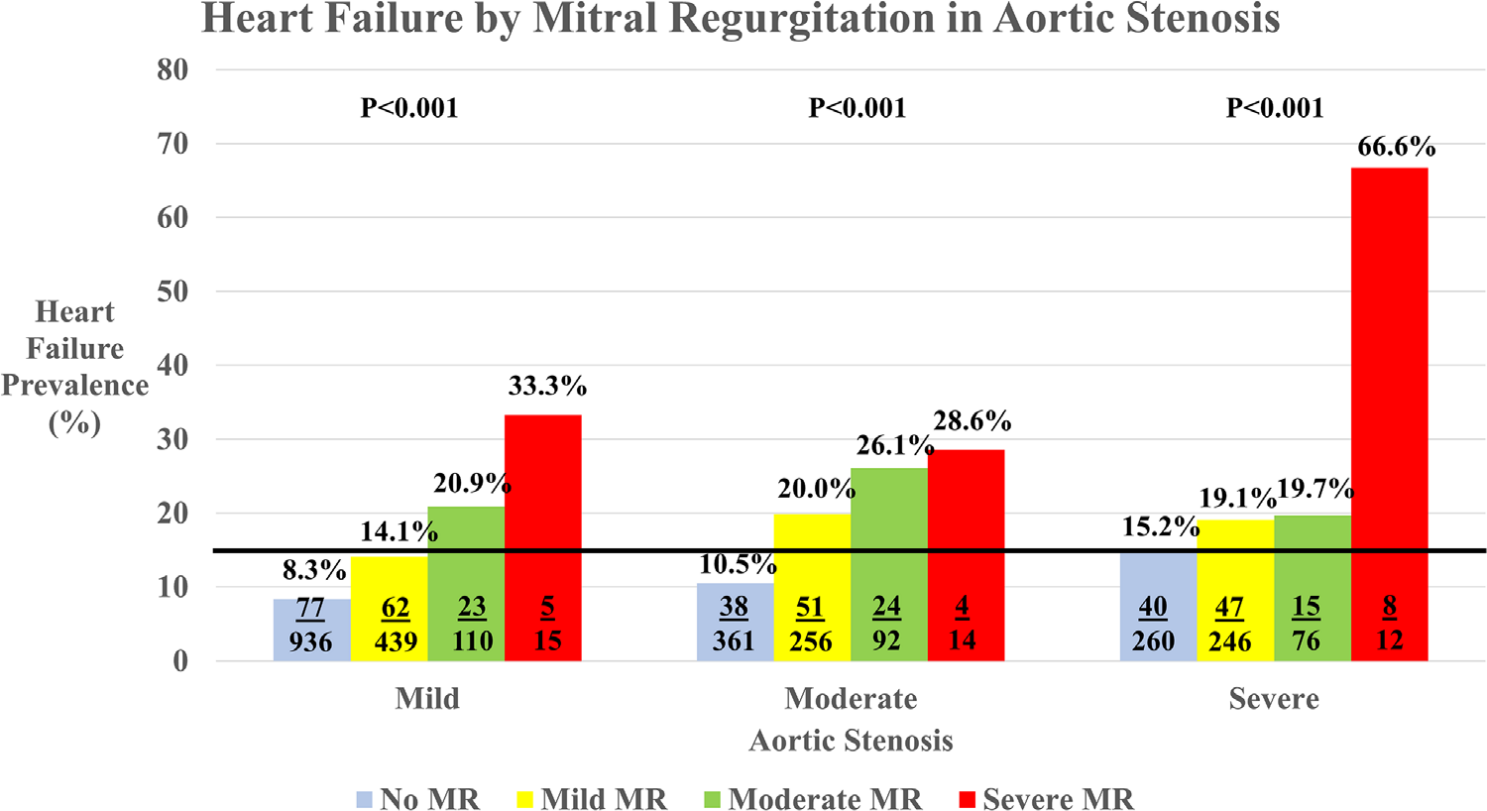
Heart failure prevalence by mitral regurgitation in aortic stenosis (AS). The p value represents testing for significant difference within the differing degrees of AS. The solid line indicates the prevalence of heart failure in severe AS without regurgitation

In unadjusted analyses, increasing AR was not statistically associated with increasing heart failure in those with severe AS, but for those with moderate AS, those with AR experienced a similar or higher prevalence of heart failure relative to those with severe AS without regurgitation (see Figure 1). However, across all degrees of AS, increasing MR demonstrated a significant statistical association with heart failure. Interestingly, those with moderate AS with moderate or severe regurgitation experienced a higher prevalence of heart failure than those with severe AS (see Figure 2). Adjusting for covariates at different levels, increasing MR had a much stronger association with heart failure compared to those with increasing AR (*p* < .0.001 vs. *p* = .313, respectively; Table 2) at all levels of adjustment.

**TABLE 2:** Results of logistic regression analysis for heart failure

Heart failure	Model I (*p*‐value)	Model II (*p*‐value)	Model III (*p*‐value)
Peak aortic pressure gradient	.044	.009	.002
Aortic valve area	<.001	<.001	<.001
Aortic regurgitation	.320	.276	.313
Mitral regurgitation	<.001	<.001	<.001
Body surface area	<.001	<.001	<.001
Race/Ethnicity	‐	.073	.448
Sex	‐	.134	.004
Age	‐	<.001	<.001
Diabetes mellitus	‐	‐	<.001
Coronary Artery Disease	‐	‐	<.001
Stroke/TIA	‐	‐	.008
Hypertension	‐	‐	.031
Chronic Kidney Disease	‐	‐	.001
Systolic blood pressure	‐	‐	.018

**FIGURE 3:**
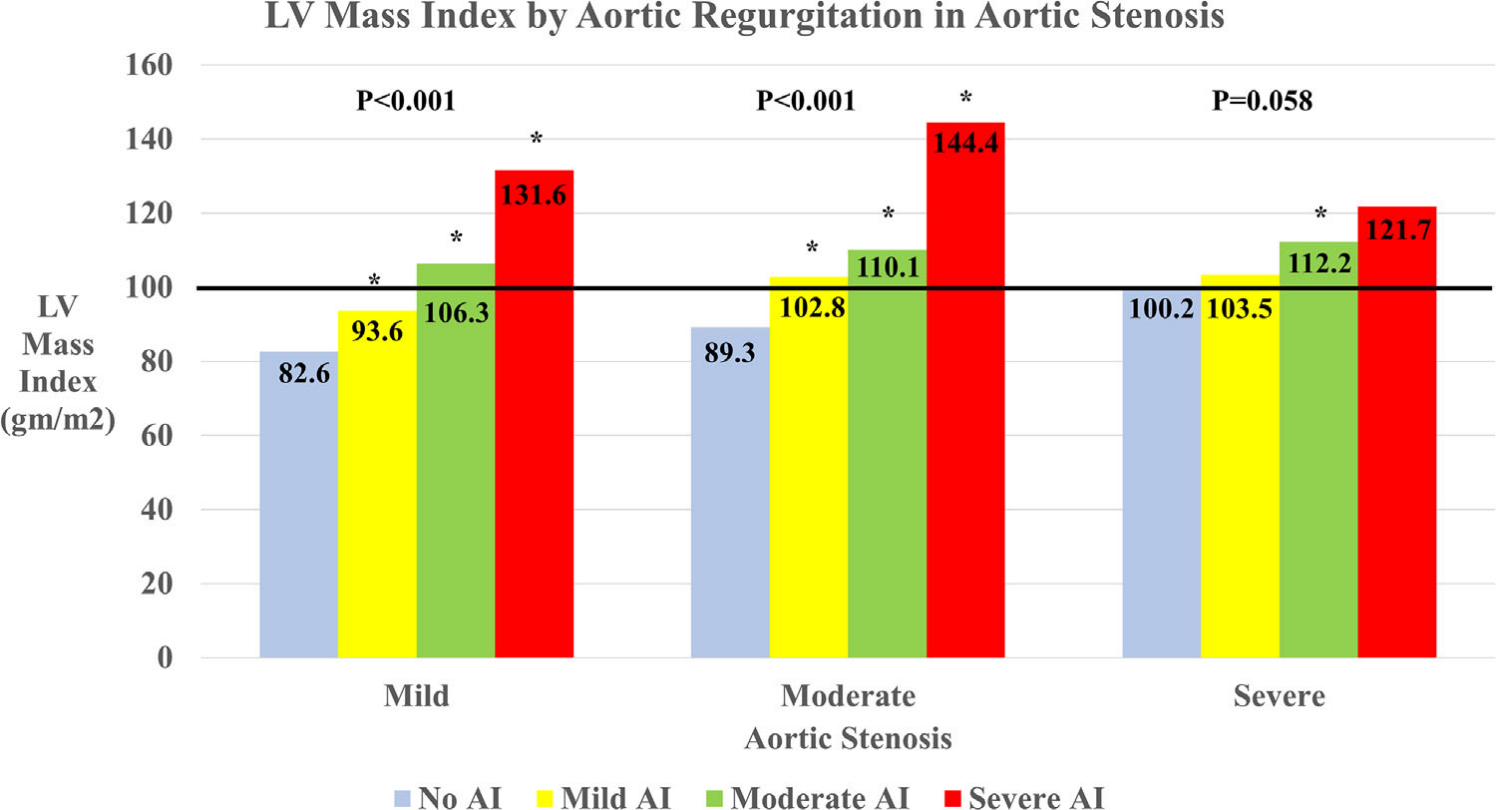
Left ventricular mass index by aortic regurgitation in aortic stenosis (AS). The p value represents testing for significant difference within the differing degrees of AS, and an asterisk (*) indicates a significant difference (*p* < .05) in comparison to those without regurgitation at that level of AS. The solid line indicates the left ventricular mass index in severe AS without regurgitation.

**FIGURE 4:**
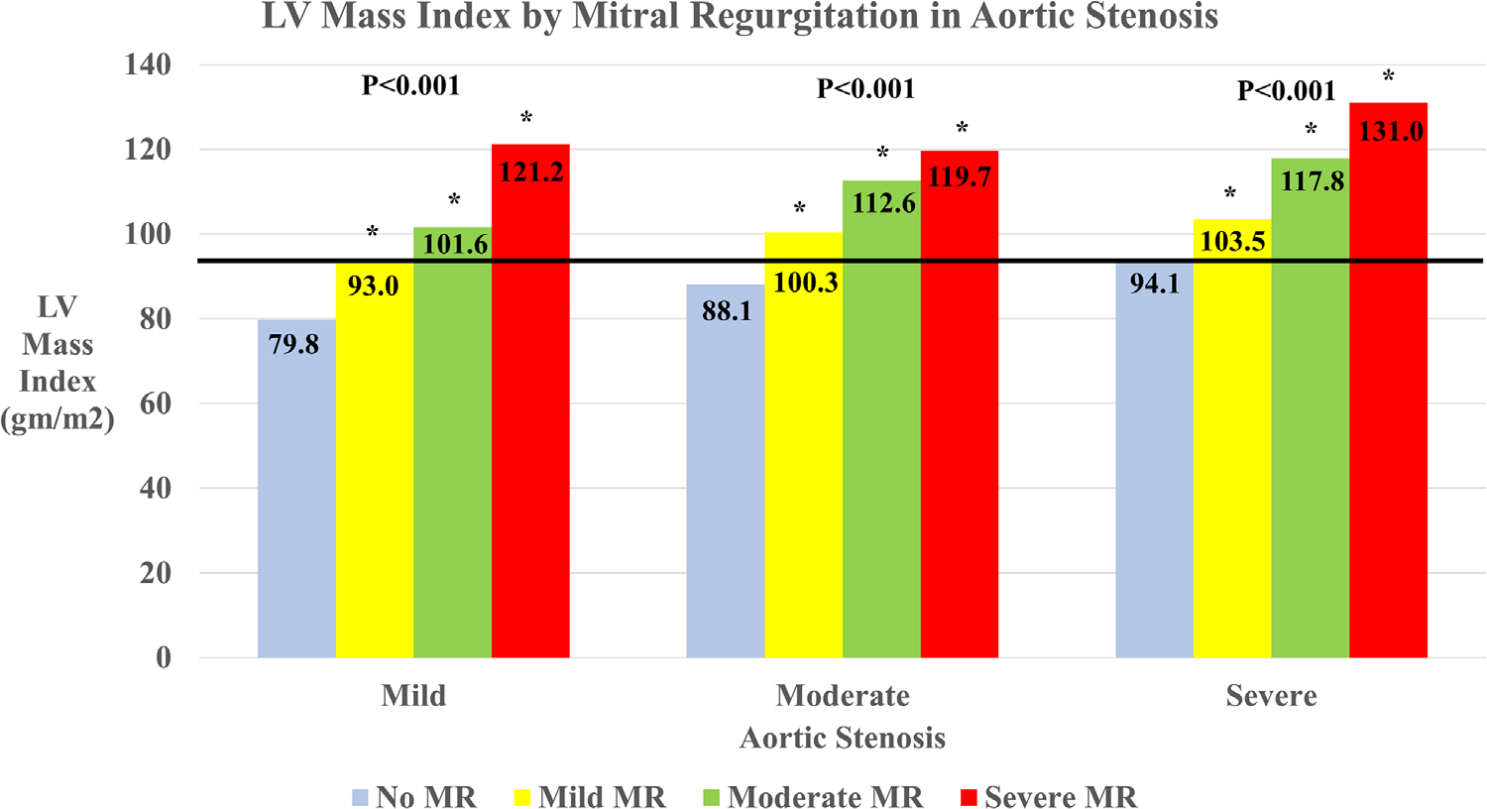
Left ventricular mass index by mitral regurgitation in aortic stenosis (AS). The p value represents testing for significant difference within the differing degrees of AS, and an asterisk (*) indicates a significant difference (*p* < .05) in comparison to those without regurgitation at that level of AS. The solid line indicates the left ventricular mass index in severe AS without regurgitation

In evaluating the potential association between valvular regurgitation and further remodeling in those with AS, both increasing aortic and MR were associated with a higher left ventricular mass index relative to those without regurgitation (see Figures 3 and 4, respectively). Overall, those with significant AS and regurgitation had a marginally higher left ventricular mass index than those with significant AS and MR. Adjusting for covariates at different levels, both increasing mitral and AR exhibited an association with increasing left ventricular mass index, even with adjustment for baseline demographics and clinical features (*p* < .0.001 vs. *p* = .007, respectively; Table 3). In those with severe AS, those with moderate or severe MR had a left ventricular internal dimension of 4.6 cm, those with moderate or severe AR had a dimension of 4.5 cm, those with no significant regurgitation had 4.4 cm (*p* = .042). As for left atrial remodeling, those with significant MR across all levels of AS saw increased left atrial volume index (*p* < .001), but in those with significant AR, only those who had severe AS experienced a relative increase in left atrial volume index (*p* = .050).

**TABLE 3:** Results of multivariate regression analysis for LV mass

LV mass index	Model I (*p*‐value)	Model II (*p*‐value)	Model III (*p*‐value)
Peak aortic pressure gradient	<.001	<.001	<.001
Aortic valve area	<.001	.010	.041
Aortic regurgitation	<.001	<.001	<.001
Mitral regurgitation	<.001	<.001	<.001
Race/Ethnicity	‐	.007	>.2
Sex	‐	<.001	<.001
Age	‐	<.001	>.2
Diabetes mellitus	‐	‐	.059
Coronary Artery Disease	‐	‐	.020
Stroke/TIA	‐	‐	>.2
Hypertension	‐	‐	<.001
Chronic Kidney Disease	‐	‐	<.001
Systolic blood pressure	‐	‐	<.001

To better understand the etiology of MR in the setting of AS, leaflet motion was used to categorize the different types of MR (see Table 4). As the severity of MR increased, so too did prolapse or flail as the etiology for the regurgitation. As the severity of AS increased, those who had MR due to restricted leaflet motion increased in parallel. In stratifying by degree of MR, 5% those with mild MR had a dilated left ventricle (LV); 8% those with moderate MR had a dilated LV; 21% those with mild MR had a dilated LV. In stratifying by the leaflet motion of MR, 4% those with normal mitral leaflet motion had a dilated LV; 14% those with either prolapse or flail mitral leaflet motion had a dilated LV; 11% those with restricted mitral leaflet motion had a dilated LV. These findings most likely reflect that those who had severe AS are also more likely to have restricted leaflets due to significant mitral annular calcification.

**TABLE 4:** Mitral regurgitation by lealet motion subdivided by severity of aortic stenosis

	Mitral regurgitation	Normal motion	Flail	Prolapse	Restricted motion
Mild aortic stenosis	Mild	96%	0	0	4%
	Moderate	81%	0	4%	15%
	Severe	44%	19%	13%	24%
Moderate aortic stenosis	Mild	95%	0	0	5%
	Moderate	83%	0	1%	16%
	Severe	50%	7%	7%	36%
Severe aortic stenosis	Mild	97%	0	0	3%
	Moderate	82%	0	1%	17%
	Severe	67%	8%	0	25%

## 4 DISCUSSION

The natural history of aortic and MR has been well‐described, but our analyses seek to describe these relationships in those with differing degrees of AS. Overall, 10.9% of those with AS also have significant MR, and 7.3% have significant AR. Several salient points may be distilled from our present analyses: (1) half of patients with AS have mixed valve disease; 16% of those with AS have at least moderate aortic or MR; (2) increasing MR has a stronger association with clinical heart failure in those with AS than those with concomitant AS and regurgitation; (3) both significant mitral and AR increase left ventricular mass in those with AS; (4) patients with moderate AS and moderate or severe MR experience higher levels of heart failure than severe AS without regurgitation.

Several pathways exist to potentially explain why heart failure was more prevalent in those with significant MR as opposed to those with significant AR. First, the presence of significant MR may be an indicator of more severe AS—the hemodynamics of which has contributed to progressive dysfunction of the mitral valve itself.^6^, ^17^ Interestingly, half of patients with significant MR at the time of transcatheter aortic valve replacement continue to have significant MR after the procedure, even after 1 year, and further, those who continue to have significant MR after transcatheter or surgical aortic valve replacement experience a higher degree of all‐cause mortality and clinical heart failure.^21^, ^22^, ^23^, ^24^, ^25^, ^26^, ^27^, ^28^, ^29^, ^30^, ^31^, ^32^ Second, in the setting of pressure overload from AS, MR unloads the ventricle directly into the left atrium and pulmonary venous circulation, which may more directly contribute to the manifestation of pulmonary edema. Third, in certain situations, the development of MR in the setting of significant AS may indicate that the LV has “burned out” and started to dilate, which may lead directly to functional MR. Lastly, those with concomitant AS and regurgitation have a slightly higher left ventricular mass index compared to those with AS and MR, which may mean that those who have concomitant AS and regurgitation experience less wall stress than those with AS and MR, and hence, they develop heart failure later.

In spite of MR having a stronger association with heart failure, both increasing mitral and AR were associated with higher left ventricular mass. Both lead to progressive left ventricular dilation from volume overload, which eventually results in clinical heart failure.^9^, ^10^, ^11^, ^12^, ^13^, ^14^ Interestingly, AR may lead to higher left ventricular mass due to added pressure overload, which may lead to lower wall stress and delay onset of clinical heart failure.^15^ Our initial thought was that this difference may be mitigated by the pressure overload from AS, but this appears not to have been the case. Mitral regurgitation itself may have offset some of the pressure overload from AS by directly unloading blood into the left atrium with more of the left ventricular mass reflecting volume overload as opposed to pressure overload. While our study population consisted of patients whose left ventricular EF was greater than 50%, there was no significant difference in left ventricular EF between those with MR compared to those with AR. Initially, significant MR may increase left ventricular EF, but with their being no difference in the left ventricular EF between mitral and AR, it may reflect that the MR may have already compromised left ventricular systolic function.

Using severe AS without any regurgitation as the standard, multiple mixed valve dysfunctions experienced similar or higher levels of heart failure. Notably, in those with moderate AS, those who also had any degree of MR experienced higher prevalence of heart failure compared to those with severe AS without regurgitation. For those with AR and moderate AS, they experienced similar rates of heart failure as those with severe AS without regurgitation, and those with moderate AS and severe regurgitation experienced even higher levels of heart failure. These observations invite several questions: (1) if a particular mixed valve combination experiences a higher degree of heart failure than isolated severe AS, should we be intervening in some of these situations sooner than what we presently are?^16^ (2) If both the mitral and aortic are involved, what would be the correct chronological order in addressing each situation? The key factor in determining when to intervene becomes the point at which the risk of waiting exceeds the risk of intervention.^33^, ^34^ Waiting to the point of heart failure or other clinical events may be too late as the development of clinical heart failure may indicate that irreversible changes have already taken place.^35^, ^36^, ^37^ Further high‐quality prospective studies are needed to provide the evidence to direct clinical care and guideline development for these cases with complex mixed valve dysfunction.

Inherent with all studies, our approach possesses limitations that should be referenced when considering our findings and conclusions. First, the echo data were derived from the clinical database of a clinical‐active tertiary referral center with a significant amount of variation in study quality, interpretation, and data reported. This approach presents several notable challenges, including quantitative classification of valvular regurgitation. While many of the echocardiographers used vena contracta, PISA, and deceleration time to evaluate regurgitation severity, because it was not entered as a measurement, we were unable to capture that information. Second, the clinical data derived from our clinical data repository from the electronic medical record represents the clinical opinion of many providers, and therefore, the clinical data lacks the precise, reproducible and accurate classification that would have resulted from prospective data judged by an adjudication committee with tight, validated clinical definitions. This most significantly impacts the data concerning heart failure where the limited documentation prevents us from being more specific and granular in our analyses and conclusions. As a crude validation, the medication use for those with heart failure are presented (see Table 5). Third, certain categories of valve dysfunction were fewer in number and may not be well described in this particular study. Lastly, by limiting our search to those with preserved systolic function, the search may only describe the initial changes associated with concomitant valve disease. Consequently, our findings and conclusions should serve as hypothesis‐generation and to justify higher quality prospective studies to better direct our clinical management in these complex situations.

**TABLE 5:** Percent medication use in those identified with heart failure

Medication class	Percent use
ACE inhibitors	58.0%
Angiotensin receptor blockers	39.4%
Beta‐blockers	93.6%
Diuretics	86.9%
Aldosterone antagonism	20.3%

## 5 CONCLUSION

In patients with aortic stenosis, nearly half have mixed valve disease with 16% having at least moderate mitral or aortic regurgitation. Further, significant mitral regurgitation has a stronger association with heart failure than significant AR, even though both increase left ventricular mass. Those with moderate aortic stenosis and significant mitral or AR experience similar or higher levels of heart failure compared to severe aortic stenosis without regurgitation. Mixed valve disease merits further studies to direct longitudinal management.

## CONFLICT OF INTEREST

None.
